# Long-Term Cognitive Effects From a Real-World Multi-Skill Learning Intervention in Older Adults

**DOI:** 10.1093/geroni/igab046.1997

**Published:** 2021-12-17

**Authors:** Leah Ferguson, Debaleena Sain, Esra Kürüm, Carla Strickland-Hughes, George Rebok, Rachel Wu

**Affiliations:** 1 University of California, Riverside, Riverside, California, United States; 2 University of the Pacific, University of the Pacific, California, United States; 3 Johns Hopkins University, Baltimore, Maryland, United States; 4 University Of California - Riverside, Riverside, California, United States

## Abstract

Previous cognitive learning interventions have focused primarily on learning one or two novel real-world skills at a time, or utilizing computer-based programs to enhance specific cognitive skills (Ball et. al 2002; Park et. al, 2014). While these studies yielded immediate cognitive improvements in participants, the long-term benefits of continuing to learn several real-world skills in older adulthood is unclear. In the present two studies, the long-term (1-year post-intervention) benefits of a multi-skill learning intervention were investigated with older adult participants. Study 1 (a pilot sample) included 6 participants (67% female, M = 66.33 years, SD = 6.41, range = 58–74 years old) and Study 2 included 27 participants (67% female, M = 69.44 years, SD= 7.12, range = 58–86 years old). Following a three month intervention which entailed simultaneously learning at least three real-world skills, such as photography, drawing, and Spanish, participants’ cognitive abilities were assessed using four tasks (Flanker, Set-Shifting, Dot Counting, and N-Back), as well as RAVLT and Digit Span. Follow-up assessments were completed at three-, six-, and 12-month follow-ups after the interventions. Linear mixed-effects regression models revealed significant cognitive improvements across time points up to one year following the intervention compared to baseline assessments. These promising results support the idea that intense learning experiences may lead to considerable cognitive growth in older adulthood, as they do earlier in the lifespan.

